# An Imbalance in the Force: The Need for Standardized Benchmarks for Molecular Simulation

**DOI:** 10.1021/acs.jcim.2c01127

**Published:** 2023-01-11

**Authors:** Kristian Kříž, Lisa Schmidt, Alfred T. Andersson, Marie-Madeleine Walz, David van der Spoel

**Affiliations:** † Department of Cell and Molecular Biology, Uppsala University, Box 596, SE-75124Uppsala, Sweden; ‡ Faculty of Biosciences, University of Heidelberg, Heidelberg69117, Germany

## Abstract

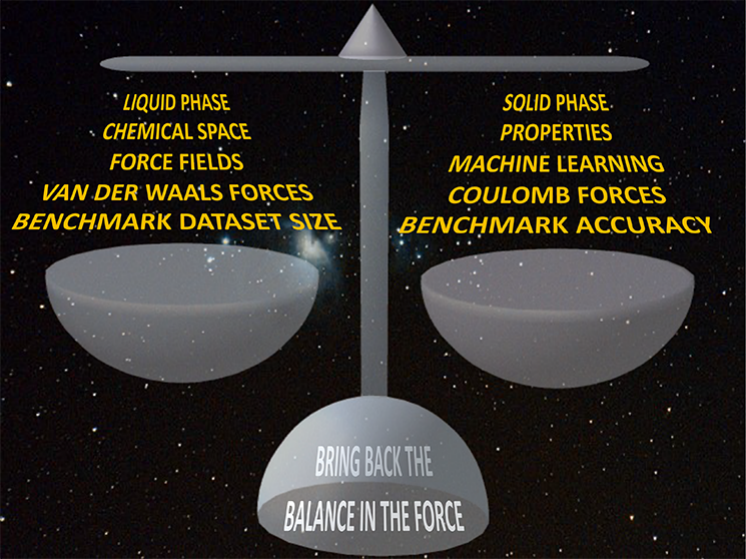

Force fields (FFs) for molecular simulation have been under development for more than half a century. As with any predictive model, rigorous testing and comparisons of models critically depends on the availability of standardized data sets and benchmarks. While such benchmarks are rather common in the fields of quantum chemistry, this is not the case for empirical FFs. That is, few benchmarks are reused to evaluate FFs, and development teams rather use their own training and test sets. Here we present an overview of currently available tests and benchmarks for computational chemistry, focusing on organic compounds, including halogens and common ions, as FFs for these are the most common ones. We argue that many of the benchmark data sets from quantum chemistry can in fact be reused for evaluating FFs, but new gas phase data is still needed for compounds containing phosphorus and sulfur in different valence states. In addition, more nonequilibrium interaction energies and forces, as well as molecular properties such as electrostatic potentials around compounds, would be beneficial. For the condensed phases there is a large body of experimental data available, and tools to utilize these data in an automated fashion are under development. If FF developers, as well as researchers in artificial intelligence, would adopt a number of these data sets, it would become easier to compare the relative strengths and weaknesses of different models and to, eventually, restore the balance in the force.

## Introduction

Over 50 years ago, Levitt and Lifson published one of the first energy calculations of a protein using a force field (FF).^1^ These authors presented their work in a modest manner, mentioning that the FF used was a “gross approximation”. Interestingly, the functional form of FFs used in most (bio)molecular simulations today is very similar to that used in the old paper. At the same time, the “real” functional form that a biomolecular FF would need is still considered unknown by some authors.^2^ Here we will not dwell on the history of FF calculations but rather refer the reader to some excellent reviews on the topic by Dauber-Osguthorpe and Hagler.^3,4^ In the second of these two reviews, Hagler describes how old lore has been forgotten and wheels reinvented. More in particular, he describes how different iterations of FF development start from different premises, adding new test systems and forgetting about older ones, which effectively leads to mending something while breaking something else.^4^ The “loyalty” to the functional form of the FF potential from the 1960s^1^ is remarkable, and Hagler is keen to point out that fixing torsion potentials cannot compensate for lacking physics in other parts of the model. Rather, he argues that more physics needs to be introduced in FFs.^4^ For example, it has been known for a long time that the description of the repulsive part of the Lennard-Jones potential^5,6^ is not very accurate and variations of the Buckingham potential^7^ are to be preferred.^8−11^

Another important issue in this respect is the addition of explicit polarization, reviewed by Jing et al.,^12^ as well as other approaches to improve the description of electrostatics^4,13^ or dispersion interactions.^14−17^ It has been shown that classical polarization models are able to capture the many-body interactions reasonably well.^18^ However, the explicit inclusion of many-body interactions, that is, going beyond the pair potential by addition of three or three and four center potentials, improves the accuracy of a FF for predicting physicochemical properties for different phases to unprecedented levels. Much effort has been gone into water, where each molecule is considered a “body” in many-body potentials.^19−22^ A somewhat more general approach was presented by Ströker et al. in a potential for argon including three-body dispersion^23^ where a single argon atom is one “body”. It is, therefore, fair to state that efforts to incorporate many-body effects are still limited to specific compounds or systems. Considerations on modeling solvents^24^ as well as routes to systematic FF design have recently been reviewed elsewhere^25^ and will not be discussed here.

Two recent reviews compare FFs for small molecules. He and co-workers focus on the evaluation of free energies of solvation,^26^ while Lewis-Atwell et al. describe how well the conformational energy landscape of small molecules in the gas phase is described by FFs.^27^ Arguably, the main aim of a general FF is to predict the properties of molecules in any environment. It can be concluded from these two reviews, however, that different FFs are needed to predict different properties. This notion is implicitly confirmed by multiple force field development teams updating their routines for generating charges to take into account multiple dielectric environments. For instance, Schauperl et al. compute atomic charges using the restrained electrostatic potential (RESP) method^28,29^ employing a polarizable continuum model (PCM) with two different dielectric constants, ε_r_, one representing water (ε_r_ ≈ 80) and one representing vacuum (ε_r_ = 1).^30^ In their method, they then use a weighted sum of the charges computed in both dielectric environments. Similarly, Bleiziffer et al. used machine learning (ML) to predict charges for small molecules based on a data set computed using PCM with a dielectric constant ε_r_ = 4, a value that is somewhere between that of vacuum and that of water.^31^ Rather than trying to generate charges that are a compromise between different environments, Hosseini and co-workers used one set of charges for a compound in aqueous solution and another one in a phospholipid membrane, when computing the potential of mean force for crossing the membrane.^32^ These examples highlight that there is an imbalance in classical force fields that makes it difficult to accurately model compounds in different environments with one parameter set, unless polarizability is treated explicitly.^12,18^ The same conclusion was drawn from benchmarks of liquids, where the enthalpy of vaporization of organic compounds (the calculation of which involves the gas-phase energy of a compound) was found to have a larger error in popular nonpolarizable force fields than pure condensed phase properties.^33,34^

Rather than using preconceived functional forms based on physically meaningful terms, ML can be used to derive a potential directly from data, using the relationship between input structure and reference values in the framework of the corresponding ML architecture.^35^ ML in chemistry has advanced rapidly, e.g., see refs (36−56), and it has been questioned whether the profession of computational chemist has a future at all in the age of machine learning.^57^ However, despite the rapid advance (or, rather, return^25^) of ML in computational modeling,^35^ the laws of physics remain valid, and these can be used to improve the accuracy of predictive models.^58^ Therefore, pursuing the holy grail of a FF that can be used in any environment remains a useful and even necessary proposition, even though the functional form for such a FF remains undecided.^2^ To speed up progress in this pursuit, the whole field of computational chemistry would do well to overcome method-compartmentalization issues by introducing common standards of reference, i.e., benchmarks with a large variety of compounds in different phases. The challenges that are suggested to be solved by using standardized benchmarks are (1) avoiding cherry-picking of properties used for validation, (2) increased applicability range of force fields, (3) objective comparison of force fields, and (4) generation of well-balanced force fields. Obviously, standardized benchmarks need to be updated and extended regularly in order to prevent tuning models for the test set rather than a training set.

We are aware that there are many force fields specialized for different kinds of systems, for instance, for clays^59^ or nanoparticles.^60^ In this perspective, we focus our discussion on evaluations and benchmarks of general FFs that treat biomolecules and/or (small) organic compounds.^61,62^ Halogen atoms need to be included since both drug compounds and pollutants^63^ interacting with biomolecules often contain these and since there is lack of relevant data for some compound classes containing halogen atoms (such as perfluorinated compounds).^64^ For reference, some of the most well-known FFs in this area are listed in Table 1. Obviously, the benchmark data sets can equally well be used for ML potentials, and indeed quite a few of the recently published data sets were developed with ML in mind as will be discussed in detail below.

**Table 1:** Some Recent Versions of Popular Force Fields

force field	Pol^a^	targets	refs
Amber ff14ipq	no	protein, RNA	(68)
Amber ff15ipq	no	protein, RNA	(69)
Amber ff14sb	no	protein, RNA	(70)
Amber ff19sb	no	protein, RNA	(71)
Amoeba	yes	protein, RNA, DNA	(72, 73)
GAFF	no	small molecules	(74)
GAFF2	no	small molecules	(75)
Charmm36	no	proteins, nucleic acids, lipids	(76)
CGenFF	no	small molecules	(77)
Charmm Drude	yes	protein, RNA	(78)
GROMOS 54A8	no	protein	(79)
GROMOS 2016H66	no	small molecules	(80)
TraPPE	no	liquids	(81)
OPLS-AA	no	liquids	(82)
OPLS3	no	drug-like small molecules, proteins	(83)
OPLS3e	no	drug-like small molecules	(84)
MM3,MM4	no	small molecules	(85)
MMFF94	no	small molecules, proteins	(86−90)
MMFF94S	no	small molecules, proteins	(91, 92)
Smirnoff99Frosst	no	small molecules	(93)
OpenFF-1.0, 1.1, 1.2, 2.0	no	small molecules	(94)
ReaxFF	yes	reactive systems	(95)

a Pol indicates whether polarizability is included in the FF explicitly.

Both FF development and ML within computational chemistry lean heavily on benchmarks from the field of electronic structure theory, because of the large amounts of data available. It should be noted, however, that the benchmarks used ultimately need to be scrutinized as well using experimental reference material.^65^ Since FF methods are computationally cheap, they allow direct comparison with condensed phase properties based on experimental data,^33,34,66,67^ circumventing potential uncertainties in quantum mechanical (QM) references.^65^ In what follows we describe both the observables relevant for evaluating FFs and existing data sets or benchmarks. We start with physicochemical properties of monomers, then move to interaction energies of dimers and complexes, before addressing the condensed phases. The perspective ends with a discussion section containing recommendations for further work on development and application of data sets.

## Monomeric Compounds

### Thermochemistry

The study of chemical reactions using QM has led to the development of multiple methods to estimate thermochemistry values, such as the Gaussian-*n* methods,^96−100^ the Weizmann methods,^101−105^ and the complete basis set model chemistry^106,107^ (not to be confused with extrapolation to the complete basis set limit discussed below). Of the properties these methods can predict, the standard enthalpy of formation Δ_f_*H*^⊖^, standard entropy *S*^⊖^, and heat capacity at constant volume *C*_V_ are particularly important for FF development. As per usual, the accuracy and computational cost of these methods vary significantly. The Gaussian-4 theory,^99^ for example, was found to be a good compromise between cost and accuracy, reaching a root-mean-square deviation (RMSD) of 11–12 kJ/mol from experimental data for Δ_f_*H*^⊖^ for 600 compounds of up to 47 atoms.^108^ Along with these method developments, benchmarks have been introduced that have been widely adopted, such as G3/05,^109,110^ W4-17,^111^ and GMTKN55.^112,113^

Reproduction of gas-phase properties such as the standard entropy *S*^⊖^ and heat capacity at constant volume *C*_V_ has been attempted using FFs as well with reasonable results^114^ based on compounds from the Alexandria library.^108,115^ FFs are known not to be very good at predicting vibrational frequencies.^114,116,117^ In the specific case of united-atom force fields, a degree of error compensation can occur when computing the standard entropy *S*^⊖^ or heat capacity *C*_V_, leading to results being quite good for the wrong reason.^80^ Vibrational frequencies are indeed difficult to predict accurately by any computational chemistry methods, which has led to the introduction of scaled frequencies in quantum chemistry. Since this is beyond the scope of this perspective, we refer to Laury et al.^118^ for an overview of frequency scaling factors. Beyond the paper mentioned,^114^ the only attempts to predict thermochemical properties from classical FFs depend on *ad hoc* empirical corrections, such as in ref (119). Hence, experimental thermochemistry data presents an opportunity for improvements of FF as well as ML potentials, although the number of compounds for which this data is available is limited to about 7000.^120^

### Molecular Structure

The lowest energy conformation of molecules, as determined experimentally or through high-level QM, provides yet another data point, since by definition the net forces on the atoms should be zero. Accurate structural data on bond lengths and covalent bond angles has traditionally been obtained from small molecule crystal structures,^121^ and for instance, Allinger and co-workers have done much work on analyzing the conformational properties of small molecules.^85,122,123^ The MM*x* FF family^85^ developed by his group remains competitive for predicting these kinds of properties.^27^ In another study, the OPLS3e^84^ and OpenFF 1.2^94^ FFs were found to be somewhat better at reproducing DFT optimized structures and conformational energies *E*_conf_.^124^ It should be noted, however, that the MM*x* FFs were optimized to reproduce experimental structures.

Aside from energy minimized structures, off-equilibrium structures are a very useful source of reference data, as they allow sampling the potential energy surface of molecules. For such structures, the forces, that are now nonzero, can be computed in addition to energies and used in model development. Structures can be prepared with MD simulations or, as is often done in case of molecular complexes (section Gas Phase Dimers and Complexes), by scaling the intermolecular distance or a relevant angle by a constant. Examples of data sets containing off-equilibrium structures will be discussed below.

### Data Sets on Monomeric Compounds

In this section, we present an overview of data sets including structures of (predominantly) monomers and corresponding energies, as well as other physicochemical properties useful for FF development (Table 3).

#### Accurate Models Require Big and Diverse Data

There are different philosophies on what kind of reference data to use, that is, experimental versus quantum chemistry and the availability of data from different sources is therefore indicated in Table 2. For instance, some force fields directly target the properties of interest, such as Gibbs energy of solvation, in their parameter development strategy.^75,125−127^ A large body of experimental data is available from handbooks, e.g., refs (128−131). The majority of data sets presented below are based on QM calculations, however. Many of these data sets were designed to train neural networks for predicting energies and forces. Therefore, these models require a substantial number of data points to capture the chemical diversity of their target system, and to make this feasible the properties are usually calculated at moderate levels of theory (mostly DFT). While FF methods contain many fewer parameters (about 1500)^25^ than neural network potentials (e.g., ≈325 000 parameters for the ANI-1ccx potential),^52^ the ML-training data sets can still be used for FF development if they cover various physicochemical properties. That being said, we limit this perspective to properties that are useful in development of halo-organic FFs, see Table 2.

**Table 2:** Some of the Most Important Properties for Development and Validation of Force Fields and Training of Neural Networks

property	symbol	source^a^	importance
Energetics			
energy relative to atoms	*E*	QM	for enthalpy of formation
excitation energy	E*	QM	for reaction kinetics in reactive models
conformational energy relative to minimum energy	*E*_conf_	QM	for intramolecular potentials
interaction energy in dimers and complexes	*E*_int_	QM	for intermolecular interactions
force vector in nonequilibrium conformations or complexes	*F*	QM	for both intra- and intermolecular interactions
vibrational frequencies	ν	B	force constants and thermochemistry, dynamics
second virial coefficient	*B*	X	gas phase intermolecular interactions
Thermochemistry			
enthalpy of formation	Δ_f_*H*^⊖^	B	validation of intramolecular interactions using experimental data
gas phase entropy	*S*^⊖^	B	for dynamics and free energy calculations
gas phase heat capacity at constant volume	*C*_V_	B	for temperature dependence of (mainly) bonded potentials
Electrostatics			
partial charges	*Q*	QM	to model electrostatic interactions
polarizability tensors	α	QM	to model polarization response
electrostatic potentials	*V*_ESP_	QM	for training electrostatic models
dipole (or higher multipoles)	μ, θ, ...	B	validation of electrostatics
Condensed Phase Properties			
lattice enthalpy	Δ*H*_latt_	X	intermolecular interactions
enthalpy of vaporization, sublimation	Δ*H*_vap_, Δ*H*_sub_	X	intermolecular interactions and phase change
density	ρ	X	intermolecular interactions
solvation free energy	Δ*G*_solv_	X	intermolecular interactions
heat capacities of liquid	*C*_V_, *C*_P_	X	temperature dependence of enthalpy
enthalpy of mixing	Δ*H*_mix_	X	intermolecular interactions
excess molar volume of mixing	Δ*V*_mix_	X	intermolecular interactions
melting point, boiling point	*T*_melt_, *T*_boil_	X	temperature dependence of intermolecular interactions and phase change
surface tension	γ	X	interface polarization and stiffness
dielectric constant	ε	X	balance between dynamics and interaction strength
viscosity and diffusion coefficient	η, *D*	X	temperature dependence of interaction strength
crystal structure coordinates and lattice parameters	*r*, *a*, *b*, *c*, α, β, γ	X	atomic radii as well as intermolecular interactions in the solid state

a The predominant source of data is indicated by either X (experiment), QM (quantum chemistry), or B (both).

Long range interactions need special consideration in the development of a ML model.^132−135^ For this purpose, data sets of interaction energies Δ*E*_int_ are particularly useful, and those are discussed in detail in the section Gas Phase Dimers and Complexes dedicated to noncovalent interactions.

It is worth noting that data sets may change over time, e.g., by adding more structures to the data set or recalculating properties at a higher level of theory. This has happened, for instance, for the GDML data set that had both an increase in number of structures and additional calculations with different levels of theory, essentially superseding its predecessor, the MD17 data set.^43,49,51,136^

#### QM*x* and Related Data Sets

There are several data sets called QM*x* (quantum mechanics *x*), where the *x* stands for the highest number of heavy atoms for that particular data set. The sources of these compounds are the generated database (GDB)-*x* data sets, that are enumerations of all possible compounds following some simple rules for chemical feasibility.^137^ QM7 is one of the earliest such large data sets.^36^ It is a subset of the organic molecules from the GDB-13 database,^137^ and it consists of seven thousand molecules containing H, C, N, O, and S. An extended version of the data set called QM7b was released later, with 13 additional properties and including chlorine atoms.^37^ Subsequently, QM8,^38,138^ consisting of ≈21 000 structures, and QM9,^138,139^ consisting of ≈134 000 structures, were introduced. QM9 includes several properties useful for FF development. Both the QM8 and QM9 data sets consist of small organic structures, in this case H, C, N, O, and F (but not Cl). The QM*x* data sets are well-known benchmarks in the field, and additions have been proposed. For instance, the QM9 data set contains additional molecules generated by the deep learning model G-SchNet^140^ and the Alchemy^141^ data set was provided to increase the number of heavy atoms from 9 to 14.

A drawback of the data sets above is that they have a limited sampling of the potential energy surfaces of the included molecules. The geometries in the QM7 data set were derived using the universal FF,^142^ and the other QM*x* data sets were minimized using DFT. This results in geometries that are at or near equilibrium only. Both ML models and FFs are limited by their training data, so in order to truly predict the energies and forces during a simulation, where the system is not at equilibrium, off-equilibrium structures are required to explore a larger conformational space. The QM7b-T^143,144^ is an example of such a data set, and it contains the same molecules as in QM7b but sampled using MD and calculated at coupled clusters level of theory. C7H10O2-17^145^ and ISO17^139,145,146^ are two additional examples that are based on the most common isomer from QM9, C_7_H_10_O_2_, and evaluated at DFT level of theory.

#### ANI-1 Data Sets

Like the QM*x* data sets, the ANI-1 data sets are also based on a GDB database, in this case GDB-11.^147^ Despite not being calculated at a very high level of theory, the original ANI-1^148^ data set consists of 22 million off-equilibrium structures of various molecules. In 2019, the same authors released the updated ANI-1x^47,149^ and ANI-1ccx^52,149^ data sets. Both of these were improved using active learning, increasing the diversity of the included conformations while bringing the total number of conformations down.^150^ ANI-1ccx was also calculated at a higher level of theory. With each of the published data sets, the authors also introduced a neural network potential trained on the respective data set presented.^42,47,52^ To benchmark the performance of their potentials, they created a new benchmarking data set, COMP6,^47^ consisting of six subsets of data. ANI-1 was later complemented by ANI-1E,^151^ to supply the data set with equilibrium structures that were not provided in the original work.

#### Other Data Sets

Various other data sets that add different chemical environments and properties to the arsenal of the FF developer are available. OE62^152^ and tmQM^153^ cover a broad range of elements. The QMugs data set contains several useful properties of biologically and pharmacologically relevant molecules extracted from the ChEMBL database.^154^ Large structural databases of molecules have also been used as a source of molecular structures for QM data sets, e.g., the TensorMol-0.1 network using molecules from ChemSpider^155,156^ and the PC9 data set based on compounds from PubChem.^44,157^ GMTKN55^113^ is a database containing 55 data sets. In addition to data sets dedicated to noncovalent interactions, in part covered in Table 4, GMTKN55 encompasses other data sets with a variety of thermochemical properties, reaction energies, and reaction barrier heights, some of which are potentially useful for ML potentials or FF development. The Alexandria library^108,115,158^ provides DFT-optimized structures of about 5000 compounds along with a range of properties such as molecular multipoles, polarizabilities, and electrostatic potentials. Yet another collection of data sets for benchmarking of QM methods is the Minnesota database by Truhlar and co-workers, which features various properties, computed or experimental where available.^159^ One such example is a data set of solvation free energies, MNSOL. The latter two databases mentioned, along with several others, have been included in ACCDB meta-database.^160^ The number of published data sets in this field is very large and describing every one in detail is out of scope of this review. However, an extensive list of data sets can be found in Table 3, and we refer the reader to the references provided there.

**Table 3:** Overview of Large Data Sets with Various Molecular Properties^a^

data set	level^b^	coverage	geometries^c^	properties	size	year	refs
QM7; QM7b	PBE0†; also ZINDO, SCS, GW	H, C, N, O, S. Up to 7 heavy atoms; incl. Cl	N/A; Opt	Δ_f_*H*; also E*, α	7165; 7211	2012; 2013	(36, 37, 137)
MNSOL	experimental	H, C, N, O, F, Si, P, S, Cl, Br, I. Neutral and charged compounds in 92 solvents	Opt	Δ*G*_solv_	790	2014	(159)
QM9	B3LYP/6-31G(2df,p), G4MP2	H, C, N, O, F. Up to 9 heavy atoms	Opt	*E*, E*, Δ_f_*H*, *S*, *C*_V_, ν, α, μ	133 885	2014	(138, 139)
QM8	CC2/def2-TZVP, TD-PBE0, and TD-CAM-B3LYP in def2-TZVP	H, C, N, O, F. Up to 8 heavy atoms	Opt	*E*, E*	21 786	2015	(38, 138)
GDML	CCSD or CCSD(T)/cc-pV(D/T)Z or PBE (vdW-TS)^43^	H, C, N, O. Small compounds.	NE	*E*, *F*	3 875 468	2017; 2019	(43, 49, 51, 136)
ISO17	PBE^145,146^ (vdW-TS)	C_7_H_10_O_2_ isomers	NE	*E*, *F*	640 982	2017	(139, 145, 146)
ANI-1	ωB97x/6-31G(d)	H, C, N, O. Up to 8 heavy atoms	NE	*E*	22 057 374	2017	(42, 148)
Yao et al.	ωB97X-D/6-311G**	H, C, N, O. ChemSpider molecules with up to 35 atoms, water clusters	NE	*E*, *F*, μ	2 979 162, 370 844	2018	(155, 156)
MPCONF196	CCSD(T)/CBS (aTZ to aQZ, extrap., ΔCCSD(T)/haDZ) or MP2-F12/aDZ, ΔDLPNO-CCSD(T)/aDZ)	H, C, N, O. Macrocycles with up to 120 atoms	Opt	*E*_conf_	13 196	2018	(162)
Alexandria Library	B3LYP/aug-cc-pVTZ, G4	Up to 4th row elements except K, Ca, incl. I. Mostly organic compounds	Opt	*E*, Δ_f_*H*, *S*, *C*_V_, ν, *Q*, α, *V*_ESP_, μ	5100	2018	(115)
COMP6	ωB97*x*/6-31G*	H, C, N, O. Six data sets, incl. drug-like compounds, peptides, and S66. Up to 312 atoms	NE	*E*, Δ*E*_int_, *F*, *Q*, μ	56 182	2018	(47)
SN2 reactions	DSD-BLYP-D3(BJ)/def2-TZVP	H, C, F, Cl, Br, I. Halide anions, methyl halides	NE	*E*, *F*, μ	452 709	2019	(163)
solvated protein fragments	revPBE-D3(BJ)/def2-TZVP	H, C, N, O, S. Fragments up to 8 heavy atoms, systems up to 120 atoms, incl. ions	NE	*E*, *F*, μ	2 731 180	2019	(163)
QM7b-T	CCSD(T0)/cc-pVDZ, other methods	H, C, N, O, S, Cl. Up to 7 heavy atoms	NE	*E*	7211	2019	(143)(144),
GDB13-T	MP2/cc-pVTZ	H, C, N, O, S, Cl. Up to 13 heavy atoms	NE	*E*	6000	2019	(143, 144)
ANI-1x	ωB97x/def2-TZVPP	H, C, N, O. Up to 63 atoms	NE	*E*, *F*, *Q*, μ	5 496 771	2019	(47, 149)
ANI-1ccx	DLPNO-CCSD(T)/CBS^52^	H, C, N, O. Up to 55 atoms	NE	*E*	489 571	2019	(52, 149)
G-SchNet data set	B3LYP/6-31G(2d,p)	H, C, N, O, F. Up to 9 heavy atoms	Opt	*E*, μ	9074	2019	(140)
Alchemy	B3LYP/6-31G(2df,p)	H, C, N, O, S, F, Cl. 9–14 heavy atoms	Opt	*E*, E*, *S*, *C*_V_, α, μ	119 487	2019	(141)
PC9	B3LYP/6-31G*	H, C, N, O, F. Up to 9 heavy atoms (singlets, doublets, triplets)	Opt	*E*, *Q*	99 234	2019	(44, 157)
Schütt et al.	PBE/def2-SVP, HF/def2-SVP	H, C, N, O. Hamiltonians and overlap matrices for conformations of several small compounds	NE	*E*, *F*	121 977	2019	(53)
OE62	PBE0† (vdW-TS), PBE0† (vdW-TS MPE–water), GW@PBE0/def2-(T/Q)ZVP	H, Li, B, C, N, O, F, Si, P, S, Cl, As, Se, Br, Te, I. Up to 92 heavy atoms	Opt	*E*, *Q*	61 489, 30 876, 5239	2020	(152)
QMspin	CASSCF(2e,2o)/cc-pVDZ-F12, MRCISD+Q-F12/cc-pVDZ-F12	H, C, N, O, F. Carbenes, triplets, singlets	Opt	*E*, ν, μ	8062, 5021	2020	(164)
tmQM	TPSSh-D3BJ/def2-SVP, GFN2-xTB^165^	H, B, C, N, O, F, Si, P, S, Cl, As, Se, Br, I. Transition metal complexes	Opt	*E*, E*, *Q*, α, μ	86 665	2020	(153)
QM7-X	PBE0† (vdW-MBD)	H, C, N, O, S, Cl. Up to 7 heavy atoms	Opt/NE	*E*, E* Δ_f_*H*, *F*, *Q*, α, μ	4 195 237	2021	(166)
ANI-1E	ωB97x/6-31G(d)	H, C, N, O. Up to 8 heavy atoms	Opt	E*, Δ_f_*H*, *S*, *C*_V_, α, μ	57 455	2021	(167)
Gastegger et al.	PBE0/def2-TZVP	H, C, O. Response properties in solvent, explicit (sampling) and implicit (PCM)	NE	*E*, *F*, α, μ	214 183	2021	(56)
BSE49	(RO)CBS-QB3^107^	H, B, C, N, O, F, Si, P, S, and Cl. Homolytic cleavage of 49 different bond types	Opt	Δ*E*	4502	2021	(168)
Guan et al.	ωB97X-V/cc-pVTZ	Potential energy surface for 19 reaction channels for hydrogen combustion	NE	*E*, *F*	361 803	2022	(169)
QMugs	ωB97X-D/def2-SVP	H, C, N, O, P, S, F, Cl, Br, I. Up to 100 heavy atoms	Opt/NE	*E*, E*, *H*, *S*, Δ_f_*H*, *S*, *C*_V_, ν, *Q*, α, *V*_ESP_, μ	2 004 003	2022	(154)
Thürlemann et al.	PBE0-D3BJ/def2-TZVP, MBIS^170^	H, C, N, O, F, S, Cl. Up to 20 heavy atoms	NE	*F*, *V*_ESP_, μ	1 013 949	2022	(171)
VIBFREQ1295	Experiment, CCSD(T)(F12*)/cc-pVDZ-F12	H, C, N, O, P, S, F, Cl, B, Si, Al	Opt	ν	141, 1295 ν	2022	(172)
Chan	CCSD(T)/CBS (W1X-2)^173^	H, C, N, O. Up to 100 atoms from NIST database	Opt	Δ_f_*H*	1500	2022	(174)

a The properties of interest are explained in Table 2.

b The † in PBE0† stands for PBE0 in FHI-AIMS,^161^ with “tight”settings/“tier 2” basis set. For the definition of CCSD(T)/CBS, see next section.

c Indicates if the geometries were optimized to reach the energy minimum (Opt) or if non-equilibrium (NE) structures were generated.

## Gas Phase Dimers and Complexes

The accurate description of noncovalent interactions is crucial for the modeling of phenomena in complex systems, such as those of interest in biology-related fields. This section presents an overview on the computational chemistry benchmark data sets dedicated to noncovalent interactions studied using dimers or multimeric complexes. Experimental data on noncovalent interactions is limited, and their reproduction by the computational methods is not straightforward.^65^ Therefore, high-level *ab initio* QM calculations are often resorted to.

### Interaction Energy

The concept of interaction energy (Δ*E*_int_) is used for the unambiguous comparison of computational methods in their capability to predict the strength of interactions. The quantity is defined as the difference between the (gas phase) energy of infinitely separated monomers (*E*_A_, *E*_B_) and that of their complex (*E*_AB_) at the distance at which monomers A and B interact. Neither zero-point vibrational energy (ZPVE) nor conformational changes upon binding are typically considered in studies mentioned below. For example, the geometries of monomers are taken directly from the minimum of the complex, without reoptimization.
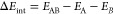
1

One of the approximations used by *ab initio* QM methods to make computations feasible is the use of truncated basis sets for the orbital description. However, calculation of the interaction energy in this way introduces a basis set superposition error, as the complex AB gains an extra stabilization compared to individual monomers A and B. There are approaches available to mitigate this artifact such as the symmetry adapted perturbation theory (SAPT) method which, however, fall outside of the scope of this perspective.^175,176^

### The Accuracy Level

For data sets featuring smaller systems, a very high level of accuracy can be reached using more demanding methods. The coupled clusters singles, doubles, and perturbative triples, CCSD(T), with extrapolation to a complete basis set (CBS) are habitually used as reference data for this purpose. This level of theory is referred to as the “gold standard” of computational chemistry.^177^ A common practice to reach this accuracy level is through a composite scheme, where the final benchmark energy is the sum of an MP2 energy in the CBS limit and a ΔCCSD(T) correlation correction to this energy. The correction is the difference between CCSD(T) and MP2 in the respective basis set. The MP2 component is extrapolated to a CBS from computations in (at least) two basis sets and the ΔCCSD(T) correlation component is then typically calculated in a smaller basis set. An example is an MP2 extrapolation from aTZ and aQZ basis sets with a ΔCCSD(T) in the aTZ basis set (here we use basis set designation where aXZ corresponds to aug-cc-pVXZ, with X being D, standing for double, T for triple, Q for quadruple, or 5 for pentuple zeta, respectively^178^). This setup, or approaches yielding comparable accuracy (see below), was deemed adequate^177^ to reach the desired “gold standard”, referred to as “gold” level from here on. Using a smaller aDZ basis set for the ΔCCSD(T) correction, which is an important factor for the accuracy, to obtain the CCSD(T)/CBS was defined as a “silver” level. Finally, going beyond the “gold” accuracy was termed the “platinum” level.^177,179^

### Is Anyone Benchmarking the Benchmarks?

The interaction energies calculated by CCSD(T)/CBS extrapolated methods were successfully tested as a part of the protocol to reproduce experimental values with errors of 0.15 to 0.3 kcal/mol for benzene–alkane clusters or 0.1 kcal/mol for small complexes of noble gases.^180,181^ Authors of these publications concluded that the experimental determination of true minima alkanes or correct computation the ZPVE were larger sources of error than the CCSD(T) calculations themselves.

Certain approximations are generally employed to render these high-level QM computations tractable. Because the use of experimental data for noncovalent interactions is often impractical, as illustrated by the two studies above, to examine the validity of these approximations, the A24 data set was created. This data set provides Δ*E*_int_ on 24 small complexes of H, C, N, O atoms with the highest accuracy available at the time. For the benchmark values, a three-point extrapolation of CCSD(T) to the CBS using large basis sets was used, with the ΔCCSDT(Q) correlation correction up to the perturbative quadruple excitations, dropping the frozen-core approximation commonly employed and additionally accounting for relativistic effects for all the elements. The approach was suggested as a “platinum” level of accuracy.^177^ The combined error due to neglecting of the following contributions (in this order of importance): coupled cluster treatment up to CCSDT(Q), correlation of core electrons, and relativistic effects was estimated to lie below 2% of benchmark interaction energy for the data set.^182^ “Gold”, using the aTZ to aQZ extrapolation with the aTZ ΔCCSD(T) term, and “silver”, using the aTZ to aQZ extrapolation and the aDZ ΔCCSD(T) term, levels yielded errors of about 1% and 2% compared to the A24 benchmark, respectively, while using the nonaugmented DZ basis set to compute the ΔCCSD(T) term results in ∼6% error.^182,183^ Regardless, both gold and silver levels of accuracy are indeed very high and definitely sufficient for applications in FF development.

### Interaction Energy Data Sets

A selection of benchmark data sets for noncovalent interactions is presented in Table 4. One of the first benchmark data set publications for noncovalent interactions featured the JSCH2005 and S22 data sets.^184^ JSCH2005 focused on nucleobases and amino acid complexes, while S22 was more general. The S66 data set, intended as an extended and more universal replacement of S22, became one of the most popular data sets for noncovalent interactions. These data sets received several updates over time, extending the coverage of potential surfaces and accuracy of the benchmark.^183,185−189^ The S66 data set of equilibrium geometries uses aTZ to aQZ extrapolation for MP2/CBS. In the latest version,^183^ the ΔCCSD(T) term is computed in haTZ, where the “h” letter for “heavy” signifies the use of the augmented basis set (with additional diffuse functions) for non-hydrogen elements only, with a minimal drop of accuracy compared to fully augmented basis set aTZ. The S101×7 data set was introduced for the development of a charge-penetration correction for the AMOEBA FF.^72^ It uses the SAPT+/CBS method for the benchmark values, which in turn were tested on S66, where the FF delivered an error of 0.16 kcal/mol.^190^ Recently, the S66 and S101 complexes have been included in the NENCI2021 data set,^191^ which broadens the coverage of elements, includes charged complexes and also an extended mapping of the potential energy surface. The potential energy surface mapping of NENCI2021 focuses, in the fashion of S101, on repulsive regions, with intermolecular distances ranging from 70 to 110% of the equilibrium distance.

**Table 4:** Overview of High-Level Benchmark Data Sets for Noncovalent Interactions^a^

data set	level	coverage	size	year	refs
S22	Gold (aTZ to aQZ, ΔCCSD(T)/aTZ)	H, C, N, O	22	2006	(184−186)
Berka et al.	Silver (aDZ to aTZ, ΔCCSD(T)/6-31G*(0.25, 0.15))	Amino acid side chains	24	2009	(209)
WATER27	Silver (aXZ; *x* = 2, 3, 4 and 5, ΔCCSD(T)/aDZ)	Water clusters, neutral and charged	27	2009	(200)
HEAVY28	(CCSD(T)/CBS^199^)	H, O, N, S, Pb, Sb, Bi, Te, Cl, Br, I	28	2010	(199)
S66; S66×8, S66a8	Gold; silver (aTZ to aQZ, ΔCCSD(T)/haTZ; aDZ^189^)	H, C, N, O	66; 66×8	2011	(183, 189, 210)
HSG	Gold (aTZ to aQZ, ΔCCSD(T)/haTZ)	Amino acid side chains	21	2011	(187, 211)
HBC6	Gold (aTZ to aQZ, ΔCCSD(T)/aTZ)	H, C, N, O. H-bonds	6 (Dis. curves)	2011	(187, 212)
Karthikeyan et al.	Gold (aTZ to aQZ, ΔCCSD(T)/haTZ)	H, B, C, N, O, F, Cl. Charge transfer	11	2011	(213, 214)
NBC10	Gold or silver CCSD(T)/CBS^187^	Benzene, pyridine, H_2_S, CH_4_	10 (Dis. curves)	2011	(187)
Mintz and Parks	Gold (aTZ to aQZ, ΔCCSD(T)/aTZ)	H, C, N, O, S. S-interactions	14×8	2012	(215)
X40; X40×10	Gold; silver (aTZ to aQZ, ΔCCSD(T)/haTZ; aDZ)	H, C, N, O, F, Cl, Br, I. Halogen bonds	40×10	2012	(216)
Granatier et al.	Gold or silver (aTZ to aQZ, ΔCCSD(T)/aTZ or aDZ)	H, C. Dispersion int.	12	2012	(217)
A24	Platinum (aTZ, aQZ, a5Z, ΔCCSDT(Q)/aDZ, core corr., relat. eff.)	H, B, C, N, O, F, Ar. Small compounds	24	2013	(182, 218)
Bauzá et al.	CCSD(T)/aTZ	H, C, N, P, S, As, Se, F, Cl, Br. Incl. ions, σ-hole interactions	30	2013	(219)
XB18; XB51	Gold (aQZ to a5Z, ΔCCSD(T)/aTZ)	H, C, N, O, P, F, Cl, Br, I, Li, Pd. Halogen bonds	18; 51	2013	(220)
S101×7	SAPT2+/CBS-scaled^190^	H, C, N, O, P, S, F, Cl, Br. Incl. ions, charge penetration	101×7	2015	(190)
Parker and Sherrill	Silver (DW-CCSD(T**)-F12/aDZ) or “Pewter” SCS(MI)-MP2/TZ	Nucleobase dimers, tetramers	∼100, ∼30 000	2015	(221)
Hostaš et al.	Silver (aTZ to aQZ, ΔCCSD(T)/aDZ)	Nucleobase–amino acids	272	2015	(222)
Temelso et al.	Silver (aDZ to aTZ, ΔCCSD(T)/aDZ), CCSD(T)-F12/DZ variants	Binding energies of water clusters	62	2015	(223)
BBI; SSI	Silver (DW-CCSD(T**)-F12/aug-cc-pV(D+d)Z)	Protein backbone–backbone; side chain–side chain	100; 3380	2017	(224)
HB375×10; IHB100×10	Gold (aQZ to a5Z, ΔCCSD(T)/haTZ) or silver (aTZ to aQZ, ΔCCSD(T)/aDZ) scaled to gold	H, C, N, O. H-bonds; incl. ions	375×10, 100×10	2020	(193)
HB300SPX×10	Gold (aQZ to a5Z, ΔCCSD(T)/haTZ) or silver (aTZ to aQZ, ΔCCSD(T)/aDZ) scaled to gold	H, C, N, O, P, S, F, Cl, Br, I. H-bonds	300×10	2020	(194)
López et al.	Gold (aQZ to a5Z, ΔCCSD(T)/aTZ)	H, C, N, O, P, S compounds complexed with Li, Na, K, Be, Mg, Ca ions	26×6	2020	(225)
R739×5	Gold (aQZ to a5Z, ΔCCSD(T)/haTZ)	H, C, N, O, P, S, F, Cl, Br, I, He, Ne, Ar, Kr, Xe. Repulsive contacts	739×5	2021	(196)
NENCI2021	Gold (aTZ to aQZ, ΔCCSD(T)/haTZ)	H, C, N, O, F, P, S, Cl, Br, Li, Na. Incl. ions	141, 7763	2021	(191)
DES370K	Gold or silver (aTZ to aQZ, ΔCCSD(T)/aQZ or aTZ or aDZ)	H, C, N, O, P, S, F, Cl, Br, I, He, Ne, Ar, Kr, Xe, Li, Na, K, Mg, Ca. Incl. ions	3691 dimers, 370 959 conf.	2021	(198)
SH250×10	Gold (aQZ to a5Z, ΔCCSD(T)/haTZ) or silver (aTZ to aQZ, ΔCCSD(T)/aDZ) scaled to gold	H, C, N, O, P, S, As, Se, F, Cl, Br, I. σ-hole int.	250×10	2022	(197)
D1200; D442×10	Gold (aQZ to a5Z, ΔCCSD(T)/haTZ); or silver (aTZ to aQZ, ΔCCSD(T)/aDZ) scaled to gold	H, B, C, N, O, P, S, F, Cl, Br, I, He, Ne, Ar, Kr, Xe. Dispersion int.	1200; 442×10	2022	(195)
Larger Systems					
L7	QCISD(T)/CBS (aDZ to aTZ, ΔQSISD(T)/631G* (0.25))	H, C, N, O. Up to ∼100 atoms	7	2013	(201)
S30	Exper. Δ*G*_a_, PW6B95-D3/QZ′ or ωB97X-D3/QZ′,^203^ HF-3c,^226^ COSMO-RS	Supramolecular host–guest complexes. Up to ∼200 atoms, ions, solvation energies	30	2015	(203)
PLF547; PLA15	MP2-F12/DZ + ΔDLPNO–CCSD(T)/aDZ; Respective PLF547 frag. benchmarks summed with B3LYP-D3/DZVP-DFT nonadditivity^204^	Amino acid–ligand. Up to ∼100 atoms. Protein–ligand. Up to ∼500 atoms	547; 15	2020	(204)
ExL8	CIM-DLPNO-CCSD(T)/CBS^227^	Large complexes. Up to ∼1000 atoms, inc. Si, Al, B	8	2021	(205)

a For the definition of levels of accuracy see the text. If multiple levels of accuracy are used by the authors, only the most accurate/recent is stated. In CBS schemes basis sets used for extrapolation and for ΔCCSD(T) correlation energy are separated by comma. (a)XZ notation stands for (aug)-cc-pVXZ basis set, where X is D for double, T for triple, Q for quadruple, and 5 for pentuple zeta, respectively. In some cases, variants of these basis sets are used, such as pseudopotential versions for heavier elements (here, we include them under the XZ notation). The haXZ signifies a use of augmented basis set for all atoms except hydrogen. For the details of computational setup, we refer the reader to the corresponding citation.

In these data sets, the “×” signifies different conformations generated from the equilibrium one by “scans”, e.g. varying the bonding angle of an energy minimum structure or scaling the intermolecular distance by defined percentages, producing points along the dissociation curve. In this manner, the coverage of the potential energy surface is often extended to include nonequilibrium geometries (see Table 4).

The Noncovalent Interaction Atlas (NCIA)^192^ is a collection of benchmarks containing specialized data sets for hydrogen bonds, e.g. neutral,^193^ charged,^193^ and neutral featuring sulfur, phosphorus, and halogens;^194^ dispersion bound complexes featuring a wider coverage of elements;^195^ nonequilibrium repulsive interactions at short distance;^196^ and σ-hole interactions.^197^ The series consistently applies the same “gold” level benchmark, and the data sets are complementary. The DES370K with about 3700 unique dimers and 370 000 points from QM optimizations, scans, and MD simulations is currently the largest data set for noncovalent interactions.^198^ There, the ΔCCSD(T) correction varied from aDZ to aQZ, depending on the systems size. The authors provide a smaller, representative subset of this data set, DES15K, and another database of about 5 million geometries with benchmark energies computed by a ML approach trained on the DES370K data set. The HEAVY28 data set, designed to serve as the benchmark data set for DFT development, is unique in that it contains more exotic heavier elements.^199^ Most of the dimer data sets neglect the deformation energies. An example of an exception is WATER27, which provides stabilization energies of water clusters and includes the energy corresponding to the conformational change upon complex formation.^200^

A few data sets are devoted to larger systems.^201−205^ Due to the size of the systems, different high-level benchmarking strategies were employed in order to deviate as little as possible from the “gold” standard. For example in PLA15, where the ligand interacts with its protein environment, the energy of individual protein fragments constituting the environment calculated at higher level is simply summed up to yield the basis for the benchmark whereas the nonadditivity is addressed at a lower level of theory (DFT).^204^ The ExL8 data set contains data on 8 different kinds of large complexes, including zeolite or boron nitride nanotubes.^205^ The computational details such as the basis set usage for this data set varied with the system size. A different take on the reference values is presented by the data set of host–guest complexes, S30. The data set provides experimental association energy Δ*G*_a_ in various solvents complemented with its computational counterpart determined with a combination of different methods.^203^

Aside from the GMTKN55 database mentioned in the section Data Sets on Monomeric Compounds and the NCIA project, other useful repositories include BEGDB,^206^ QCArchive,^207^ and the Computational Chemistry Comparison and Benchmark DataBase.^208^

### Force Field Approaches to Complexes

**Figure 1:**
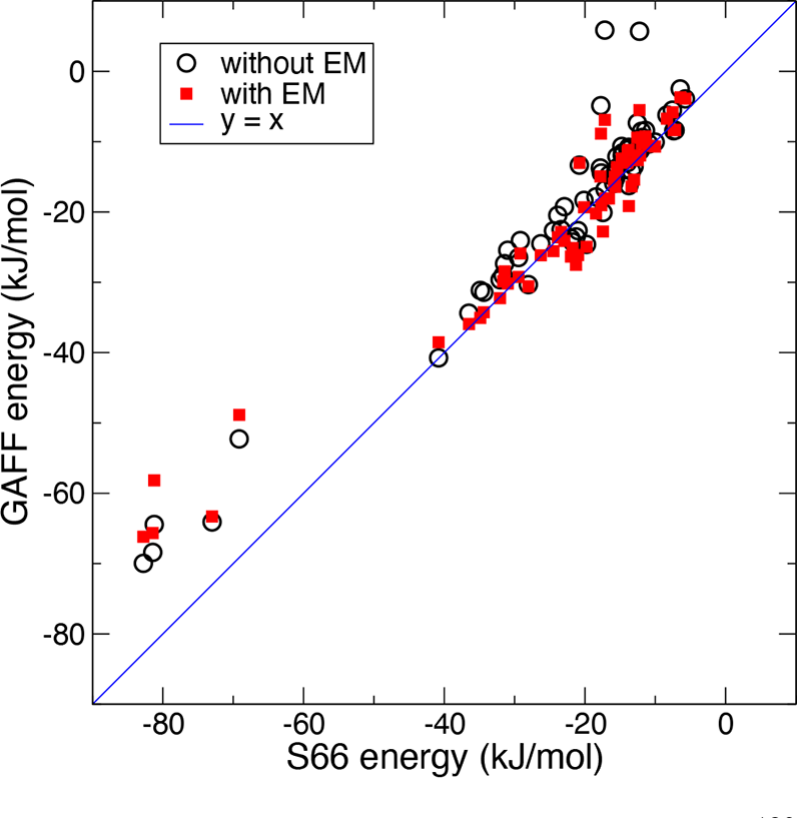
Correlation for interaction energies with S66 as reference^189^ and GAFF as target. For raw data, see Table S1. EM stands for energy minimization. Statistic without EM, *r*^2^ = 95.8%, slope *a* = 0.85; with EM, *r*^2^ = 96.2%, slope *a* = 0.91. Slope computed from a fit to *y* = *ax*.

**Figure 2:**
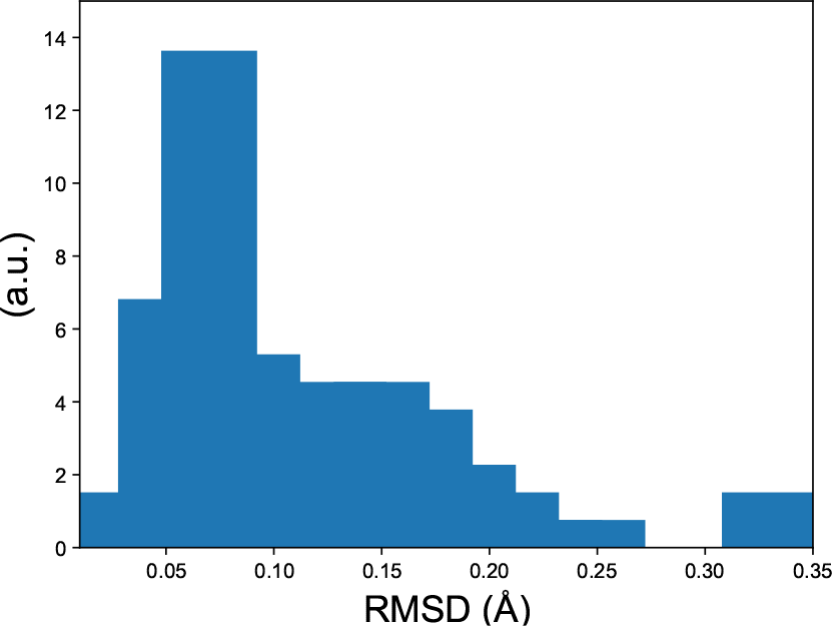
Root mean square deviation from S66 dimer coordinates after force field energy minimization. For raw data, see Table S1.

In order to illustrate how existing QM data sets can be used to validate FFs, we present a comparison between the FF interaction energies and benchmark energies of S66^228^ (Table 4). The FF dimer interaction energies were computed according to eq 1, see Methods for details. The coordinates were taken directly from the data set (Figure 1, “without EM”) and, in addition, from the result of a FF energy minimization of the input geometries (Figure 1, “with EM”). This was done since the FF energy minimum usually does not correspond to the QM energy minimum. Table S1 lists the energies of the complexes from QM as well as from the FF before and after minimization. It also lists the RMSD of the coordinates. Figure 2 shows the distribution of RMSD after minimization, to evaluate whether the structures remain well-conserved in the FF. Such comparisons give quantitative information about the performance of a FF and by analysis of its accuracy for different chemical compound classes detailed clues for improvement of FF models can be derived. The structures in the S66 data set have been used earlier to investigate electrostatic interactions by FF methods,^229^ as well as for the development of models for charge penetration.^190,230,231^ In addition, the S66×8 data set (Table 4) has been used for FF development by Vandenbrande and co-workers.^232^

## Condensed Phases

### Organic Crystals

A large amount of experimental data is available for the condensed phases that can be used for both developing and evaluating FFs. Hagler was one of the first to take advantage of molecular crystals for this purpose,^233,234^ structures for which can be found in the Cambridge Structural Database.^235^ Here, we ignore the related but specialized field of crystal structure prediction (reviewed recently here^236^) and focus on MD simulation studies on molecular crystals. The obvious advantage of crystals over liquids is that the position of the atoms is known (in most cases). This means that the properties of the models can be evaluated with more certainty than in the liquid phase,^4^ that is, if the crystal structure is preserved in the FF simulation. Price and co-workers note that the weak intermolecular forces in some crystals put high demands on FFs.^237,238^ Although it has been shown that special-purpose models can reproduce specific crystal polymorphs,^239^ this is not generally possible. Indeed, Nemkevich et al.^240^ conclude that molecular simulation of crystals can give qualitative results at best. The often small intermolecular forces in organic crystals may lead to changes in the relative orientation of compounds within the crystal, or even (partial) melting, if the forces are not well-balanced.^241^ A further issue with simulations of crystals is that it may be cumbersome to evaluate many different crystals since it is nontrivial to assess whether a system is in the crystalline phase and, if so, what polymorph it is. In addition, some crystals are in a plastic crystal phase at certain conditions, that is the molecules occupy fixed lattice positions, with some more or less restrained degrees of freedom.^241,242^ Some computational benchmarks based on organic crystals have been proposed (Table 5). Reilly and Tkatchenko extended the C21 database^243^ to form the X23 database,^244^ both studying molecular crystals using DFT. The X23 database has been evaluated using FFs by Nyman et al.^245^ and by Teuteberg et al. using combined quantum mechanics/molecular mechanics (QM/MM).^246^ Bernardes and Joseph^248^ studied 18 drug-like aromatic compounds by performing experiments to evaluate the enthalpy of sublimation Δ_sub_*H* and then compared the results to FF simulations. Apart from the enthalphy of sublimation, they also study the relative stability Δ_trs_*H* of crystal polymorphs. Finally, Schmidt and co-workers studied 30 crystals of small organic molecules using long MD simulations,^241^ and apart from Δ_sub_*H* they determined melting temperatures *T*_melt_ and solid densities ρ.

**Table 5:** Computational Chemistry Benchmarks Using Organic Crystals^a^

data set	properties	MAE (Δ_sub_*H*)	method	size	year	ref
C21	Δ_sub_*H*	4.8	PBE and better	21	2012	(243)
X23	Δ_sub_*H*	3.9	PBE and better	23	2013	(244)
X23	Δ_sub_*H*, lattice	9.2	FIT^247^	23	2016	(245)
X23	Δ_sub_*H*	11.7	QM/MM	23	2019	(246)
Bernardes and Joseph	Δ_sub_*H*, ρ, lattice	5.5	OPLS/AA^126^	18	2015	(248)
Schmidt et al.	Δ_sub_*H*, ρ, *T*_melt_	6.4	GAFF^74^	27	2022	(241)

a Size of the data set is given as well as the mean absolute error (MAE) of the best method for predicting Δ_sub_*H* (kJ/mol) in the corresponding study. Lattice parameters are indicated by “lattice”, solid density by ρ.

Although all these papers use different data sets and computational methods, it is tempting to compare the accuracy for predicting Δ_sub_*H*. The most accurate DFT used was PBE0^249^ with many-body dispersion correction,^250^ yielding a MAE of 3.9 kJ/mol.^244^ Bernardes and Joseph published a FF MAE of 5.5 kJ/mol,^248^ whereas Schmidt et al. find 6.4 kJ/mol;^241^ both of these used different data sets than X23, however, which means the numbers are not directly comparable. Data and inputs for the work presented by Schmidt et al. are available on GitHub.^251^

### Organic Liquids

Historically, it was the study of (organic) liquids that has laid the foundation for MD simulations.^24,252^ Many properties of bulk liquids have been measured over the last hundred or so years, and the results are available in databases and handbooks.^128−131^ Since classical simulations of neat liquids are not very demanding in terms of computer resources, the available experimental data on liquids can be used to both derive^82,253,254^ and evaluate FFs. Indeed, a paper by Caleman et al. scrutinizing FFs by computing seven bulk properties for about 150 liquids^33^ found several systematic flaws, in particular for surface tensions and dielectric constants. As a result, a series of other papers has been published with the aim of addressing these shortcomings, using the data provided by Caleman et al.^33,255^ as a reference, e.g. for the improvement of the OPLS-AA FF^256^ and the GROMOS 2016HH FF^80^ and evaluation of the TRaPPE FF.^257^ One important conclusion from follow-up papers,^66,258,259^ is the recommendation to apply explicit long-range dispersion interactions using the particle-mesh Ewald algorithm for Lennard-Jones potential (LJ-PME).^260−262^ The surface tension (vacuum–liquid) is particularly affected by these interactions^66,258^ (for a review on surface tensions, see ref (263)), but also systems as simple as liquid carbon dioxide.^259^ Based on these lessons, LJ-PME was used in melting simulations of organic crystals.^241^ Whether the use of LJ-PME is advantageous for simulations of biomolecules with current force fields is less clear.^264^ Simulation studies of Gibbs energy of solvation of organic compounds in organic liquids were performed by Zhang et al.^34,67^ based on earlier empirical work by Katritzky and co-workers.^265−267^ These results were used in FF development and evaluation^268−270^ as well as for comparison in FF based predictions of water–octanol partition coefficients.^64,271^ It is clear that organic compounds are excellent diagnostic tools for FF development.^114^ Nevertheless, due to the biological relevance of proteins, nucleic acids, and phospholipids, the community has put much more effort in evaluating biomolecular FFs for these kinds of molecules, e.g., see refs (272−282). Experimental reference data on thermophysical and thermochemical properties of (organic) liquids and liquid mixtures can be found, for example, in the NIST ThermoML Archive repository.^131^ It contains various experimental data of complex systems–up to ternary mixtures. It has been expanded to include metals and their compounds, too, and recently encompassed about 8 000 000 data points.^283,284^ This subtopic is so large, that it would warrant a specialized review, and we will therefore not discuss it further here. Likewise, there is an enormous body of work on solvation in water, that has been reviewed elsewhere.^24,285,286^

## Discussion

Enrico Clementi and his group pioneered force field development based on artificial intelligence and databases of QM data as early as the 1990s.^25,287,288^ In modern times, the Parsley (Open) FF is perhaps a good example of a FF to be largely derived from QM calculations and (in part) validated based on liquid properties.^94^ Although its functional form remains true to classic force fields,^1^ systematic improvements using the ForceBalance algorithm^8^ suggest that further accuracy gains may be possible. Interestingly, the Parsley Open FF and other well-known FFs were benchmarked^289^ using data from the QCArchive.^207^ Given the modeling target defined in the Introduction, that is halo-organic compounds plus biologically relevant inorganic compounds, what does the perfect data set look like? Is there a difference between data sets for model development and for benchmarking? An ideal data set would consist of enough structures to capture all of the diversity of the modeling target. Furthermore, relevant physicochemical properties have to be present (Table 2) and, for quantum chemical data sets, calculations for each and every structure should be carried out using a sufficiently high (*ab initio*) level of theory. The data set has to be easy to access and to work with, for instance, with a simple to use application programming interface such as in ref (284). Importantly, the amount of independent data points should be much larger than the amount of parameters to be optimized, which is a few thousand for classical force fields^25^ but much larger for neural networks.^52^ From the monomer databases listed in Table 3, both the Alexandria Library^231^ and QMugs^154^ cover all the targeted chemical elements (and some more) and provide at least nine properties relevant for model development per compound (Table 2). In contrast, OE62^152^ supports all the target elements but only two properties. One of the properties especially advantageous for the development of models with accurate electrostatics is the electrostatic potential of a molecule. Both the Alexandria Library^115^ and QMugs^154^ provide this, but the number of compounds is limited to about 5000 for the former while the basis set used for the DFT calculations is relatively small for the latter, which may lead to poor predictions of polarizability.^115,290−292^ Although both these data sets contain frequencies, which hold information on the curvature of the potential energy surface, they do not contain energies for off-equilibrium structures, which are needed to go beyond the harmonic approximation. A number of data sets mentioned in Table 3 have nonequilibrium structures but always with a limited coverage of the chemical elements (see section Recommendations for Data Sets). Swann and coauthors proposed to use machine learning to create a data set as a way to enforce diversity. Rather than picking representative compounds manually, their automated selection is based on molecular descriptors to cover the chemical space in an unbiased manner.^293^ Data sets containing noncovalent interaction energies are particularly useful for evaluating and developing long-range interactions in force fields as well as ML models. DES370K is the largest data set for this purpose, making it suitable for training of ML methods.^198^ The data sets in NCIA are smaller and dedicated to specific interaction types (Table 4). However, their advantage is that their benchmarks are at the same, consistent level of theory, which means that energies can be compared directly between data sets, and the data sets are complementary. The consistent level of theory is especially important for ML methods.^35^ Considered as a whole, the NCIA to date contains 2206 unique dimers with 5 or 10 points along dissociation curves (Table 4). Of the interaction databases, both DES370K^198^ and NENCI2021^191^ cover all the target elements. The NCIA^192^ also has a very broad coverage but does not yet feature complexes with inorganic ions. These, for example, can be found in a data set by López.^225^ Data sets covering less common elements include HEAVY28^199^ and the Exl8 data set of large complexes.^205^ If a more compact data set is desirable, the S66 benchmark data set is already well-established, offering comparison with older benchmarking studies.^183,189,210^ The use of standardized benchmark data sets (or their combinations) has several major advantages. First, it allows for consistent and straightforward comparison between different force fields. Improvements of a newly developed force field over its predecessors will be more clearly apparent if both are compared to the same benchmark data. It would prevent adding of new test systems while forgetting about the original ones and later “reinventing the wheel” situations, as alluded to in the Introduction and discussed in ref (4). Similarly, with standardized benchmark testing, it would be easier to see how well does a parametrization targeted to reproduce one specific property fare for other properties. That is, what trade-offs do researchers accept when adopting new methods or parameters? Thus, the habitual testing against multiple standardized benchmarks might encourage the development of balanced FFs with more general applicability. In practice, it can be expensive, and sometimes plain impossible, to calculate physicochemical properties for large compounds or clusters at a high level of theory. As a result there usually is a trade-off between more accurate calculations using a high level of theory (dimers and complexes in Table 4) and more data points (monomeric compounds in Table 3). The more computationally expensive a data set is to prepare, the more important it is that is made accessible following the FAIR (Findable, Accessible, Interoperable, Reusable) principles,^294^ not least to reduce the carbon footprint of the field.^54^ Work toward making experimental data more FAIR is underway as well.^284^ We argue here, that a higher level of theory is needed for benchmarking than for model development. At the end of the day, models need to be validated against experimental data or the highest level quantum chemistry that is available. However, there still are large amounts of information in accurate DFT calculations. If one is aware of potential pitfalls,^115^ some of the large data sets in Table 3 can be used for model development. It should be added, that experimental data is not flawless either. Besides direct experimental uncertainty and conflicting numbers, errors may be introduced when values are manually transferred from (old) papers into large databases. For instance, during the development of the Alexandria library, a list of close to 200 errors in databases of physicochemical properties was collected (Table S2 in ref (108)). To find the inadequacies of the models, such as a FF lacking essential physics in its functional form,^4^ it is recommended to test models outside their “comfort zone” as well. For instance, how is the model affected by a change in temperature (and/or pressure), and taking it a step further, how does the model perform in different phases (gas, liquid, solid)? Or, if a model has been tuned to predict bulk properties well, how reasonably are interface properties described? If the focus was on kinetic properties, such as diffusion coefficients, how well are energy-related properties captured?^295^ If a model was derived for equilibrium structures, how well are off-equilibrium structures described? In any case, after extending the model to better fit the problematic cases, it should be retested again on the original training set, if the general improvement of the model is to be sought.^4^ In order to develop and validate FFs, condensed phase simulations have to be performed. In the case of small molecule force fields that are intended as, e.g., protein ligands, it may be sufficient to compute free energies of solvation in liquids, including water.^24^ For general force fields it is possible, albeit costly, to optimize FFs using liquid simulations by slowly varying parameters until experimental observables are reproduced.^9,253,254,296−299^ In this manner, a few parameters at a time can be tuned, against one or more observables.^300^ In addition, inherently parallel methods such as the Multistate Bennett Acceptance Ratio method can be used to speed up parametrization of FFs for single compounds in the condensed phase.^301,302^ Oliveira et al. proposed a framework to optimize a force field for a limited class of compounds with shared parameters at once using, e.g., the liquid density and enthalpy of vaporization as the reference.^303,304^ Extension of such methods to a complete halo-organic force field remains to be done. If all else fails, scientific contests may be used to separate the wheat from the chaff. The Critical Assessment of Structure Prediction was one of the first such contests, where researchers were given amino acid sequences with the task to predict a protein structure.^305,306^ Although force fields have successfully been used to predict three-dimensional structures of small proteins,^307^ the computational cost is way too large to use protein structure prediction for force field development and benchmarking, although many partial comparisons exist.^272,273,308^ The Industrial Fluid Properties Simulation Collective aims for researchers to predict properties of liquids and solvated compounds.^309^ Properties such as the viscosity of alcohols and surface activity of compounds, that are difficult to predict, have been addressed in these challenges. Unfortunately, this challenge seems to be dormant at the time of writing. The Statistical Assessment of Modeling of Proteins and Ligands (SAMPL) challenge is still very much active, however.^310,311^ It is a competition revolving around drug design but also more fundamental molecular properties such as distribution constants have been targeted. Such blind tests, where as of yet unpublished experimental data is the target of predictions, help to evaluate and diagnose models. Properties of pure liquids are readily accessible and therefore useful for evaluating force fields.^33,66,131,258,312^ The data sets used by Caleman et al. are available^255^ for reuse by other groups. Possible topics for blind tests would be prediction of excess properties of mixing two liquids,^94,313,314^ or the Gibbs energy of solvation in non-water solvents.^34,67^ The relatively poor performance of force fields for the prediction of octanol–water partition coefficients shows there is room for improvement.^64,271^ Another potential target would be prediction of free energy of association of compounds in different solvents.^315^

### Recommendations for Data Sets

The coverage of data sets presented in this perspective is extensive, and great progress has been made in recent years to increase the amount, quality, and accessibility of the data sets. For force fields targeting (halo)organic compounds, there is still room for improvement of data and tools. For instance,

- Broadening of chemical diversity,^293^ in particular compounds containing phosphorus and sulfur at different valence states.
- Keeping in mind the importance of nonequilibrium structures with corresponding energies and forces.
- Benchmarking of more properties per compound, such as the electrostatic potential.
- Increasing the coverage of interaction energies (and forces) for complexes of neutral compounds with inorganic or molecular ions.
- The simultaneous use of the points above in data set construction or the use of comparable level of theory/consistent methodology in such a way that data sets may be used together.
- More ready-to-use properties of liquids like those available on the Virtual Chemistry Web site.^255^ These can be used for validating force fields by comparing to, e.g., the ThermoML database.^131^ Addition of liquid mixtures would provide a useful resource as well.

### Final Words

MD simulations of crystals highlight that there indeed is an imbalance in the forces predicted by force field models.^237,238,240,241^ This imbalance can manifest itself in, for instance, changes in the unit cell shape or incorrect energies or melting points.^241^ Similar problems have been described in simulations of organic liquids^33,34,66,67^ and gas phase studies by force fields.^114,124^ To improve on this in a systematic manner, it would be good if the force field community would adopt a number of the data sets described in this perspective. Addition of new data sets is still needed to cover particular areas of chemical space but much can be done with the existing data sets already. For the development of classical force fields as well as ML models it would be advantageous to

1. Perform training and testing including single molecule properties such as conformational energies, vibrational frequencies, and electrostatic properties. Examples of data sets targeting these properties are listed in Table 3.
2. Compare FF predictions of interaction energies to values of high-level benchmark data sets (in a way exemplified in Figures 1 and 2). Examples of such data sets are listed in Table 4. This may contribute to a more consistent and transferable evaluation of FFs. The NCIA and DES370 are particularly relevant.
3. Include the experimental properties of condensed phases in the analysis of performance, such that the transferability to bigger and more complex systems of interest may be improved.

## Methods

Energy calculations were performed using the GROMACS software^316^ (version 2021) using the generalized Amber FF (GAFF).^74^ Molecular topology files were downloaded from the Virtual Chemistry Web site,^255^ based on the paper on thermochemistry calculations using FFs.^114^ No cut-offs were used for Coulomb and van der Waals interactions. The dimer coordinates were taken from the S66 database.^210^ The energy of all dimers and their constituting monomer was calculated using GROMACS. For the potential energy of minimized systems, the molecules were energy minimized until the potential energy had converged. Energies as well as the root-mean-square deviation of coordinates are given in Table S1.

## Data Availability

Input topologies corresponding to Figure 1 are available from github.^251^

## Supporting Information Available

Table containing interaction energies corresponding to Figure 1 and coordinate RMSDs for dimers from the S66 data set (PDF)
