# Supplementary material for: An Imbalance in the Force: The Need for Standardized Benchmarks for Molecular Simulation
Source: J Chem Inf Model. 2023 Jan 11;63(2):412–31. doi: 10.1021/acs.jcim.2c01127 (PMC9875315; doi:10.1021/acs.jcim.2c01127)
Supplement: Supplementary file 1 — ci2c01127_si_001.pdf [file ci2c01127_si_001.pdf]

An Imbalance in the Force: The Need for Standardized Benchmarks for Molecular Simulation. Supporting Information.

Kristian Kříž, Lisa Schmidt, Alfred T. Andersson, Marie-Madeleine Walz, and David van der Spoel*

Department of Cell and Molecular Biology, Uppsala University, Box 596, SE-75124 Uppsala, Sweden

E-mail: david.vanderspoel@icm.uu.se

Phone: +46 18 471 4205

Table S1: Dimerization energes (kJ/mol) computed using the GAFF force field[1] compared to gold standard quantum chemistry from the S66 data set.[2] Energy before and after energy minimization (EM), and root mean square deviation (Å) of coordinates after minimization. GAFF input files taken from the Virtual Chemistry website.[3,4]

Complex	QM	before EM	after EM	RMSD
01-water-2	-20.97	-22.64	-26.12	0.154
02-water-meoh	-23.85	-20.47	-23.60	0.173
03-water-menh2	-29.44	-26.47	-29.24	0.091
04-water-methylacetamide	-34.39	-31.42	-34.26	0.137
05-meoh2	-24.48	-22.67	-25.57	0.127
06-meoh-menh2	-32.07	-29.65	-32.26	0.065
07-meoh-methylacetamide	-34.88	-31.16	-35.06	0.210

08-meoh-water	-21.28	-23.55	-27.52	0.114
09-menh2-meoh	-13.02	-13.52	-15.41	0.119
10-menh2-2	-17.67	-14.46	-19.04	0.083
11-menh2-methylacetamide	-22.93	-19.24	-24.12	0.192
12-menh2-water	-30.97	-25.44	-30.18	0.062
13-methylacetamide-meoh	-26.28	-24.52	-26.16	0.143
14-methylacetamide-menh2	-31.64	-29.04	-29.87	0.135
15-methylacetamide-2	-36.49	-34.40	-35.93	0.324
16-methylacetamide-water	-21.75	-24.28	-25.13	0.054
17-uracil-2-BP	-73.00	-64.08	-63.30	0.088
18-water-pyridine	-29.18	-24.07	-25.90	0.044
19-meoh-pyridine	-31.42	-27.35	-28.44	0.032
20-acoh-2	-81.23	-64.44	-58.17	0.150
21-acnh2-2	-69.14	-52.28	-48.85	0.039
22-acoh-uracil	-82.77	-69.97	-66.18	0.153
23-acnh2-uracil	-81.45	-68.40	-65.66	0.078
24-benzene-2-PIPI	-11.40	-8.40	-9.26	0.232
25-pyridine-2-PIPI	-15.90	-15.93	-15.97	0.066
26-uracil2	-40.80	-40.75	-38.53	0.088
27-benzene-pyridine	-13.98	-12.98	-13.02	0.067
28-benzene-uracil	-23.40	-22.52	-22.84	0.056
29-pyridine-uracil	-28.04	-30.34	-30.58	0.060
30-benzene-ethene	-5.71	-3.92	-3.88	0.017
31-uracil-ethene	-13.93	-11.13	-11.38	0.048
32-uracil-ethyne	-15.45	-12.11	-13.63	0.080
33-pyridine-ethene	-7.55	-5.52	-5.82	0.064
34-pentane-2	-15.75	-14.54	-15.01	0.058

35-neopentane-pentane	-10.90	-10.32	-10.58	0.057
36-neopentane-2	-7.38	-8.44	-8.36	0.019
37-cyclopentane-neopentane	-10.03	-10.05	-10.70	0.039
38-cyclopentane-cyclopentane	-12.49	-11.46	-12.64	0.102
39-benzene-cyclopentane	-14.70	-11.98	-12.43	0.045
40-benzene-neopentane	-11.92	-10.29	-10.28	0.030
41-uracil-pentane	-20.13	-18.35	-19.33	0.070
42-uracil-cyclopentane	-17.12	-16.78	-18.07	0.067
43-uracil-neopentane	-15.43	-13.72	-13.99	0.059
44-ethene-pentane	-8.34	-6.21	-6.73	0.034
45-ethyne-pentane	-7.18	-8.38	-8.34	0.032
46-methylacetamide-pentane	-17.81	-13.74	-14.94	0.135
47-benzene2-TS	-11.83	-9.38	-9.70	0.053
48-pyridine2-TS	-14.67	-11.59	-12.50	0.067
49-benzene-pyridine-TS	-13.78	-10.78	-11.17	0.062
50-benzene-ethyne-CHpi	-11.95	-8.52	-9.39	0.067
51-ethyne2-TS	-6.44	-2.50	-3.69	0.118
52-benzne-acoh-ohpi	-19.77	-24.62	-24.95	0.152
53-benzene-acnh2-nhpi	-18.43	-17.84	-20.21	0.313
54-benzene-water	-13.76	-16.24	-19.16	0.139
55-benzene-meoh-ohpi	-17.44	-20.07	-22.78	0.102
56-benzene-menh2-nhpi	-13.38	-14.19	-16.36	0.115
57-benzene-peptide-nh-pi	-21.99	-23.76	-26.34	0.177
58-pyridine2-CHN	-17.73	-4.88	-8.86	0.151
59-etyhne-water	-12.25	5.70	-5.51	0.212
60-ethyne-acoh-ohpi	-20.78	-13.35	-13.03	0.058
61-pentane-acoh	-12.16	-11.27	-11.95	0.070

62-pentane-acnh2	-14.76	-10.68	-12.43	0.115
63-benzene-acoh	-15.68	-15.27	-16.42	0.053
64-methylacetamide-ethene	-12.56	-7.34	-9.42	0.140
65-pyridine-ethyne	-17.17	5.87	-6.90	0.187
66-menh2-pyridine	-16.59	-14.72	-18.08	0.107

References

(1) Wang, J.; Wolf, R. M.; Caldwell, J. W.; Kollman, P. A.; Case, D. A. Development and Testing of a General AMBER Force Field. *J. Comput. Chem.* **2004**, *25*, 1157–1174.

(2) Řezáč, J.; Riley, K. E.; Hobza, P. S66: A Well-balanced Database of Benchmark Interaction Energies Relevant to Biomolecular Structures. *J. Chem. Theory Comput.* **2011**, *7*, 2427–2438.

(3) van der Spoel, D.; van Maaren, P. J.; Caleman, C. GROMACS molecule & liquid database. *Bioinformatics* **2012**, *28*, 752–753.

(4) van der Spoel, D.; Ghahremanpour, M. M.; Lemkul, J. Small Molecule Thermochemistry: A Tool For Empirical Force Field Development. *J. Phys. Chem. A* **2018**, *122*, 8982–8988.
